# Bolus Residue Scale: An Easy-to-Use and Reliable Videofluoroscopic Analysis Tool to Score Bolus Residue in Patients with Dysphagia

**DOI:** 10.1155/2015/780197

**Published:** 2015-11-12

**Authors:** Nathalie Rommel, Charlotte Borgers, Dirk Van Beckevoort, Ann Goeleven, Eddy Dejaeger, Taher I. Omari

**Affiliations:** ^1^Neurosciences, ExpORL, KU Leuven, 3000 Leuven, Belgium; ^2^Gastroenterology, Neurogastroenterology and Motility, University Hospitals Leuven, 3000 Leuven, Belgium; ^3^Translational Research Center for Gastrointestinal Diseases (TARGID), KU Leuven, 3000 Leuven, Belgium; ^4^Radiology, University Hospitals Leuven, Leuven, Belgium; ^5^ENT, Head & Neck Surgery, MUCLA, University Hospitals Leuven, Leuven, Belgium; ^6^Geriatric Medicine, University Hospitals Leuven, Leuven, Belgium; ^7^School of Medicine, Flinders University, Bedford Park, Australia; ^8^The Robinson Research Institute, University of Adelaide, Adelaide, Australia

## Abstract

*Background*. We aimed to validate an easy-to-use videofluoroscopic analysis tool, the bolus residue scale (BRS), for detection and classification of pharyngeal retention in the valleculae, piriform sinuses, and/or the posterior pharyngeal wall.* Methods*. 50 randomly selected videofluoroscopic images of 10 mL swallows (recorded in 18 dysphagia patients and 8 controls) were analyzed by 4 experts and 6 nonexpert observers. A score from 1 to 6 was assigned according to the number of structures affected by residue. Inter- and intrarater reliabilities were assessed by calculation of intraclass correlation coefficients (ICCs) for expert and nonexpert observers. Sensitivity, specificity, and interrater agreement were analyzed for different BRS levels.* Results*. Intrarater reproducibility was almost perfect for experts (mean ICC 0.972) and ranged from substantial to almost perfect for nonexperts (mean ICC 0.835). Interjudge agreement of the experts ranged from substantial to almost perfect (mean ICC 0.780), but interrater reliability of nonexperts ranged from substantial to good (mean 0.719). BRS shows for experts a high specificity and sensitivity and for nonexperts a low sensitivity and high specificity.* Conclusions*. The BRS is a simple, easy-to-carry-out, and accessible rating scale to locate pharyngeal retention on videofluoroscopic images with a good specificity and reproducibility for observers of different expertise levels.

## 1. Introduction

In patients with dysphagia, pharyngeal bolus residue is a significant predictor of postswallow aspiration [1, 2]. Residue is the result of incomplete bolus clearance due to poor propulsion, weak pharyngeal vigor, and/or impaired upper esophageal sphincter (UES) relaxation [2, 3]. As a result, this bolus residual material poses an aspiration risk as it may enter the airway after swallowing. A higher risk for postswallow aspiration is expected with increased volume of the residue, because a larger amount of retention will overflow the boundaries of the available space [2]. In particular, pharyngeal bolus residue is most commonly located in the valleculae and/or the piriform sinuses [4].

To date, the gold standard to detect postswallow residue in a clinical setting is a videofluoroscopic swallow study (VFS). In order to evaluate those VFS recordings, several qualitative and quantitative methods have been developed to evaluate pharyngeal retention.

First, on a VFS image, pharyngeal residue can be rated using qualitative, also called observational, methods. Dejaeger et al. classified pharyngeal residue into one of four categories based on residue presence and location: no residue, residue in valleculae, residue in piriform sinuses, and residue in both locations (diffuse) [5]. This approach is limited due to the lack of information about the amount of residual material [6]. Hence, an alternative manner to rate pharyngeal residue is to use an ordinal scale. An example of such a scale is Hind's three-point ordinal scale. This scale estimates the amount of residue (with 0 = no residue; 1 = coating of residue; 2 = pooling of residue) at various locations such as the oral cavity, valleculae, posterior pharyngeal wall, piriform sinuses, and the upper esophageal sphincter (UES) [7]. Also, Rosenbek et al. described an equivalent scale with 0 = no residue, 1 = minimal residue, and 2 = moderate-to-substantial residue [8]. Yet, these ordinal rating scales are limited by lack of specific cut-off score for minimal or moderate-to-substantial residue or pooling [6]. An example of a semiquantitative method is the scale of Han et al. (2001). They rated pharyngeal residue as a percent-filled space by assigning four grades based on perception of the amount of residue in comparison to the width of the valleculae [6, 9]. A “0” grade represents no residue, “1” refers to <10% filling of the width of the valleculae, “2” refers to 10–50%, and “3” refers to >50%. In addition, Eisenhuber et al. developed a 1–3 grading scale to score the amount of residue in the valleculae or the piriform sinuses [2]. Grade “1” is mild bolus residue and corresponds to <25% of the height of the valleculae or the piriform sinuses filled with residue. Grades “2” and “3” represent moderate (25–50% of the height) and severe (>50% of the height) pharyngeal retention in the valleculae or piriform sinuses.

Apart from observational methods, a few quantitative analysis methods for the measurement of the area of residue on radiographic images have been suggested. Two recently published methods are the Vallecular Residue Ratio Scale (VRRS) [10] and the Normalized Residue Ratio Scale (NRRS) [6].

This paper presents an easy-to-use observational method developed to rate the presence or absence of bolus residue. The aim of the current paper was to describe inter- and intrarater reliability as well as sensitivity and specificity of this simple method to measure pharyngeal residue on lateral videofluoroscopy images of swallowing.

## 2. Methods

### 2.1. Study Database Swallows and Selection

Fifty 10 mL bolus swallows were randomly selected from a master database of bolus swallows recorded in 30 patients and 10 control subjects who had undergone videofluoroscopy under the aegis of clinical research protocols approved by the Research Ethics Committee (S51993-B32220097615), University Hospitals Leuven, Belgium. The order of individual master database swallows was randomized and 50 were consecutively selected comprising 30 dysphagic patient swallows and 20 control swallows. The 30 randomly selected patient swallows were from 18 patients (12 male, mean 64 yrs, range 13–95 yrs) of whom twelve had a neurological history (7: stroke, 1: Parkinson's disease, 2: dementia, 1: postneurosurgery, and 1: neuromuscular disorder), 1 had postcervical surgery, and 5 had unknown etiologies at the time of study. The 20 randomly selected control swallows were from ten subjects (3 male, mean 37 yrs, range 24–47 yrs). The number of swallows selected for each patient/control ranged from one to four (11 with 1 swallow analyzed; 8 with 2 swallows; 4 with 3 swallows; 3 with 4 swallows).

Controls had no swallowing difficulties nor other symptoms suggestive of a motility disorder.

As per routine clinical fluoroscopy, test boluses were administered orally via syringe. Boluses were standardized across all patients and controls studied. A standard liquid contrast material (MicropaqueH) was given as liquid bolus and used with thickener (Thick & Easy) for semisolid bolus test conditions. A low osmotic hydrosoluble iodinated contrast agent (UltravistH) was used when aspiration was suspected. The corresponding videofluoroscopy files of all selected swallows were compiled into a study database for sequential analysis by observers.

### 2.2. Fluoroscopy Analysis

Video-loops of the fluoroscopic images of swallows were acquired at 25 frames per second. Each observer performed repeat BRS analyses of deidentified video-loops edited such that only one bolus swallow was displayed per video-loop.

**Figure 1:**
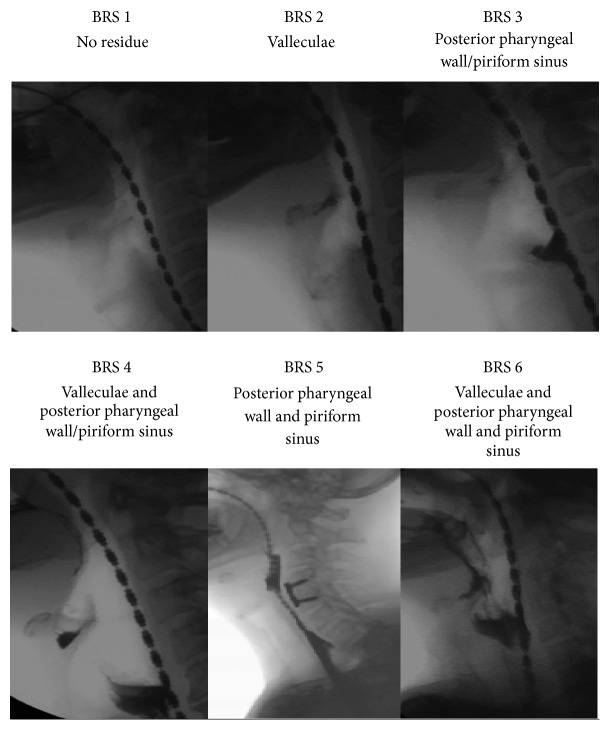
Videofluoroscopic image for each BRS score (1–6).

The BRS scored for the presence or absence of postswallow residue in the valleculae, piriform sinuses, and/or posterior pharyngeal wall. A bolus residue scale (BRS) score between 1 and 6 according to the number of structures showing evidence of residue was assigned: no residue in any of these structures was assigned a BRS score of 1. If residue was present, then additional scores were weighted towards the anatomical regions in which residue posed an aspiration risk (Table 1). A higher BRS score is more severe and corresponds to a higher risk of aspiration because the location is closer to the airway. A BRS score of 4–6 was considered highly clinically significant as indicative of residue on at least two structures. Examples of videofluoroscopic images for different BRS levels are shown in Figure 1.

### 2.3. Observers

Ten observers with ranging experience were asked to participate in the study. Four observers were considered to be “experts” in fluoroscopy as they routinely reviewed fluoroscopy images (geriatrician, radiologist, and two speech pathologists); six were considered informed nonexperts (a medical student, a research assistant, two nurses, and two gastroenterology trainees). All observers received identical training in the BRS. Reference material, demonstration videos, and practice swallows were provided to allow the observers to develop competence in the BRS before proceeding to their formal analysis of the database swallows. Each observer performed repeat analyses of all swallows in their own time.

### 2.4. Statistical Analysis

Intrarater test/retest reproducibility and interrater reliability of the BRS were assessed by calculation of intraclass correlation coefficients (ICCs). For intrarater reproducibility, data derived during the first and second analyses were correlated for each observer. For interrater reliability, data derived from the first analysis were correlated for each combination of observers.

An expert consensus BRS score was determined for each swallow based upon the most frequently assigned BRS score for that swallow as determined by the experts (if two BRS scores were equally frequently assigned, the average was taken). The degree of agreement between the expert consensus score and individual experts/nonexperts was determined for the different levels of BRS (BRS 2+ to 6) by using Cohen's kappa statistic (*κ*), weighted kappa that corrects for the effect of chance and bias [11]. The interrater agreement between the expert consensus score and individual scorings of expert and nonexpert observers compares BRS scores greater than a specified cut-off consensus score. As a result, any observer scoring greater as the cut-off score agrees. For example, when a cut-off consensus of 3 (BRS 3+) is chosen, then all observer gradings higher than BRS 3 (BRS 4–6) agree with the consensus. The interpretation for ICC and *κ* values is as follows: 0.00 = no agreement, 0.00–0.2 = slight (poor), 0.21–0.40 = fair, 0.41–0.60 = moderate, 0.61–0.8 = substantial (good), and 0.81–1.00 = almost perfect (very good) [12]. Prognostic value was also assessed through calculation of sensitivity and specificity.

## 3. Results

Complete repeat scorings were returned by all observers. Inter- and intrarater ICCs for individual experts and nonexperts as well as expert consensus scores are shown in Table 2. The intrarater reproducibility of the BRS was almost perfect for both experts (mean ICC 0.972, range 0.895–1.000) and ranged from substantial to almost perfect for nonexperts (mean ICC 0.835, range 0.716–0.987). To evaluate the degree of agreement of nonexperts with experts, the ICC for expert consensus score versus nonexpert scoring of the BRS was calculated. Nonexperts seemed to be less reliable in detecting residue compared to experts with the ICC ranging from moderate to almost perfect (mean ICC 0.719, range 0.533–0.834). The interrater ICCs were as expected highly variable as presented in Table 2. Because of the less reliable intrarater reproducibility of the nonexperts, interrater ICCs between individual nonexperts were not computed. Interjudge agreement between expert observers ranged from substantial to almost perfect (mean ICC 0.780, range 0.716–0.880) (Table 2).

The cross-classifications of the scores given by the experts and the nonexperts are shown in Tables 3 and 4, respectively, as well as the total frequencies of the assigned scale scores for the first and second grading by both experts and nonexperts. The diagonal in both tables represents the pattern of agreement (i.e., identical scores) between the first and second grading per judge. The experts gave an identical score in 187 of 200 replicate gradings, which corresponds to an agreement percentage of 94%. The nonexperts had an agreement percentage of 75% (225 of 300 replicates gradings). When both experts and nonexperts did not assign an identical score on both gradings, a score within 1 unit (3.5% and 9.7%, resp.) or 2 units (2% and 9%, resp.) was given as second score.

**Figure 2:**
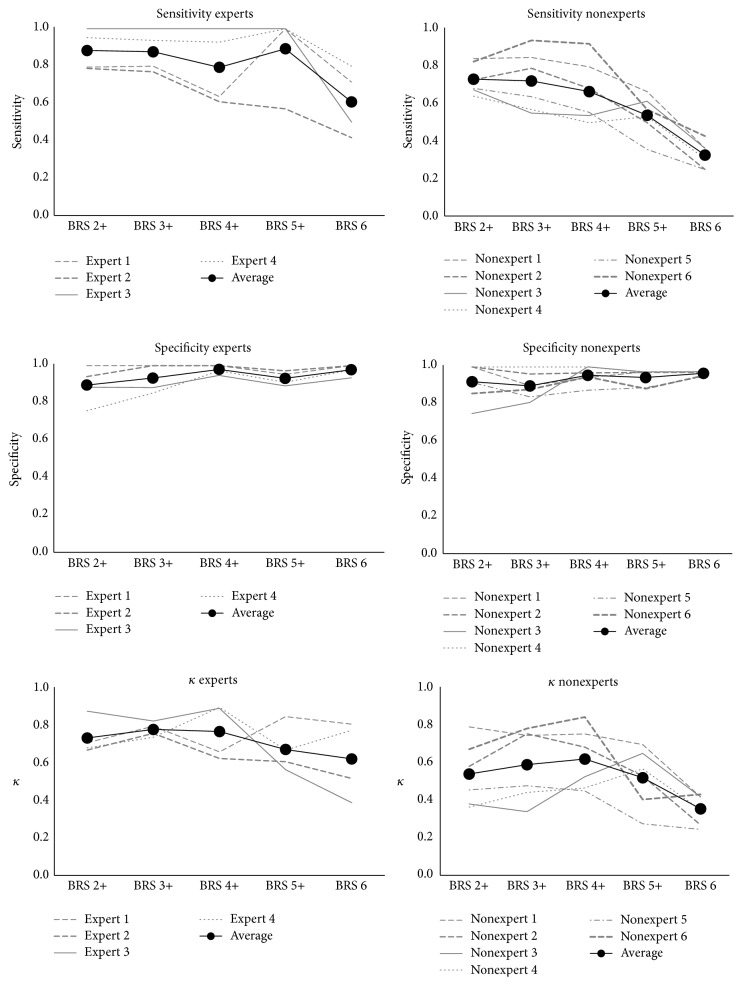
Specificity, sensitivity, and agreement (*κ*) of nonexperts and experts with the expert consensus score in relation to different bolus residue scale scores.

The sensitivity and specificity as well as the kappa-coefficients for every grading of bolus residue are shown in Figure 2 for expert and nonexpert observers. The kappa-coefficients compare the amount of agreement between a single observer (expert/nonexpert) and the expert consensus for different levels of the BRS score (i.e., a cut-off score). In the same way, sensitivity, specificity, and average kappa-coefficients on each BRS level are displayed in Table 5 for both experts and nonexperts. A substantial agreement was observed between expert scoring and expert consensus for different BRS levels. However, this agreement could be expected since expert observers showed good inter- and intrarater reliability. Expert scoring of any residue (2+) and clinically significant residue (4+) agreed substantially with the expert consensus (mean *κ* 0.737, sens. 0.88, spec. 0.89 and 0.731, sens. 0.79, and spec 0.98, resp.). In contrast, nonexpert scoring revealed higher variability on different BRS levels. In detecting the presence of any residue (BRS 2+), nonexpert scoring agreed moderately with the expert consensus score (mean *κ* 0.543, sens. 0.73, and spec. 0.92). With a view to determine the presence of clinically significant residue (BRS 4+), nonexpert scoring agreed substantially with the expert consensus score (mean *κ* 0.623, sens. 0.67, and spec. 0.96), although individual agreement ranged from *κ* 0.452 (moderate) to *κ* 0.847 (almost perfect). A low reliability was observed in detecting clinical significant residue in all structures (BRS 6) (mean *κ* 0.36, sens. 0.97, and spec. 0.33).

## 4. Discussion

The bolus residue scale (BRS) is an observational scale to determine the absence or presence of residue in the valleculae, the piriform sinuses, and/or the posterior pharyngeal wall. To evaluate whether this scale can be used as a reliable tool to grade residue, the reproducibility and reliability of this radiological-based method in both expert and nonexpert observers were assessed in this study. Fifty fluoroscopic images were repeatedly scored by four experts and six nonexperts by assigning a grade ranging from 1 to 6 according to the anatomic structures in which the residual material was located. The BRS appeared reproducible in the hands of different observers. The intra- and interrater reproducibility of experts were almost perfect. In fact, our experts were fairly unanimous, which makes the BRS a reliable instrument for clinical use. The less experienced observers in radiological assessment obtained poorer results compared to expert observers. Interrater reliability between nonexperts and experts was rather moderate, as there was a large variability between individual nonexperts. Additionally, the agreement on both gradings was more variable for nonexperts than experts (75% versus 94%). For both experts and nonexperts, nonidentical gradings differed by only one or two units. Moreover, experts agreed well in detecting any residue (BRS 2+) and clinically significant residue (BRS 4+) locating in 1 of the 3 locations. Nonexpert observers showed a substantial agreement with the expert consensus scoring in detecting any residue (BRS 2+) or clinically significant residue (BRS 4+). Interestingly, in both groups, a larger variability on BRS 5+ and BRS 6 was observed, indicating that it may be more difficult to rate residue which affects more than one anatomical site. Presumably, a related difficulty could be to differentiate pooling from coating. Pooling of bolus material is seen as any material that is present in the pharynx or larynx cavities before and/or after swallowing [13]. Coating is the condition where bolus residue only moistens the pharyngeal walls [14].

BRS scoring of nonexperts has a high specificity for both BRS 2+ score (any residue) and BRS 4+ score (clinically significant residue). However, both BRS 2+ and BRS 4+ showed low sensitivity (0.73 and 0.67, resp.), indicating that a large proportion of the population will potentially be undetected (false negative) or that residue severity will be underestimated. For experts, both sensitivity and specificity were higher for 2+ and 4+. These findings support the use of the BRS to screen for patients with pharyngeal retention by trained clinicians. Hereby, at-risk patients can be referred for further investigation and diagnosis, thereby preventing further pulmonary complications and chronic undetected dysphagia. Our data show however that nonexperts (such as nurses, researchers, and medical students) can but need to be trained to reliably judge fluoroscopic images with respect to pharyngeal residue grading.

Several quantitative and qualitative analysis methods have been developed to evaluate pharyngeal retention performed on videofluoroscopic recordings. Advantage of software-based techniques such as NRRS or VRRS is their quantitative nature and high interrater reliability, but a limitation of these methods for routine clinical practice is that they require extra handling of the VFS data for analysis [10]. As a result, those methods can be time consuming. Therefore, we believe an efficient and easy-to-use method like the BRS can be useful due to the low handling complexity of the scale.

Besides quantitative and observational methods, semiquantitative methods have been developed. Those methods take into account severity as well as the amount of residue. Although these semiquantitative ordinal scales were designed to improve the accuracy of residue detection, it is well accepted that they have limited precision and they showed poor reliability [15]. The BRS does not rate the volume of the residue as it may be inaccurate to estimate a 3D volume on a 2D VFS image.

It is important to emphasize that the BRS is a qualitative assessment. Counting this potential limitation, Omari et al. however reported a significant correlation between the BRS and an objective nonradiological marker of clinically relevant postswallow residue, called the integrated nadir impedance to impedance ratio. This metric is an objectively derived parameter for bolus residue using impedance manometry recordings [3]. Furthermore, it is assumed that increased postswallow residue, marked as a higher BRS score, will be associated with a higher risk for aspiration. This assumption is based on the fact that the areas covered with bolus residue (according to the BRS score) are closely located at the airway entrance. Omari et al. confirmed this suggestion by correlating BRS scores with the Swallow Risk Index (SRI). SRI is an objective metric correlating to aspiration which is calculated using four pharyngeal pressure flow parameters [16]. In summary, objective evidence is emerging that the BRS outcome is linked not only to detection of bolus residue but also to aspiration in patients with dysphagia.

## 5. Conclusion

In summary, this paper described the validation of a new observational scale, the bolus residue scale (BRS), to detect and classify bolus residue in the valleculae, piriform sinuses, and/or posterior wall of the pharynx. This study explored the reliability and reproducibility of this method as well as sensitivity and specificity for both expert and nonexpert observers. The bolus residue scale seems to have a good specificity and reproducibility for different types of observers. The study shows that even nonexperts showed a good but a more variable agreement with lower sensitivity than experts. Hence, the BRS showed to be a reliable instrument that can be used in the clinical setting by professionals experienced in evaluating radiological swallow studies. The BRS is a simple, easy-to-carry-out, and accessible analysis method to rate and locate pharyngeal retention. In clinical practice, the BRS can be used to indicate the severity of pharyngeal residue as a quantifiable score.

## Figures and Tables

**Table 1:** Bolus residue scale (BRS) scores according to the number of structures affected by residue.

BRS score	Indication of residue
1	No residue
2	Residue in valleculae
3	Residue in posterior pharyngeal wall *or* piriform sinus
4	Residue in valleculae *and* posterior pharyngeal wall *or* piriform sinus
5	Residue in posterior pharyngeal wall *and* piriform sinus
6	Residue in valleculae *and* posterior pharyngeal wall *and* piriform sinus

**Table 2:** Intra- and interrater test/retest reproducibility for expert and nonexpert observers assessed by calculation of intraclass correlation coefficients (ICC).

Observer	Intrarater ICC	Interrater ICC	Expert consensus
Expert 1	0.997	**0.780**	0.880
Expert 2	0.895		0.820
Expert 3	1.000		0.893
Expert 4	0.997		0.915

Nonexpert 1	0.789	**—**	0.834
Nonexpert 2	0.716		0.752
Nonexpert 3	0.796		0.639
Nonexpert 4	0.926		0.732
Nonexpert 5	0.797		0.533
Nonexpert 6	0.987		0.823

Average experts	**0.972**	**—**	**0.877**
Average nonexperts	**0.835**	**—**	**0.719**

**Table 3:** Cross-classifications of both gradings given by 4 experts are calculated for 50 swallows: the pattern of agreement (diagonal) and the total frequency of assigned scores for gradings 1 and 2 are shown.

	Grading 2						Total	%
	1	2	3	4	5	6		
Grading 1								
1	**85**	3	0	0	0	0	88	44
2	1	**25**	0	1	1	1	29	14.5
3	0	0	**10**	1	1	0	12	6
4	0	0	0	**40**	0	1	41	20.5
5	0	0	0	1	**0**	1	2	1
6	0	0	0	1	0	**27**	28	14

Total	86	28	10	44	2	30	200	100
%	43	14	5	22	1	15		

**Table 4:** Cross-classifications of both gradings given by 6 nonexperts are calculated for 50 swallows: the pattern of agreement (diagonal) and the total frequency of assigned scores for gradings 1 and 2 are shown.

	Grading 2						Total	%
	1	2	3	4	5	6		
Grading 1								
1	**70**	6	5	5	0	2	88	29.3
2	5	**44**	2	7	0	4	62	20.7
3	1	3	**23**	2	0	2	31	10.3
4	1	2	1	**28**	3	7	42	14
5	0	0	0	2	**1**	4	7	2.3
6	0	4	1	5	1	**59**	70	23.3

Total	77	59	32	49	5	78	300	100
%	25.7	19.7	10.7	16.3	1.7	26		

**Table 5:** Averaged intraclass kappa (*κ*), sensitivity, and specificity for experts and nonexperts by scale scores.

	BRS 2+	BRS 3+	BRS 4+	BRS 5+	BRS 6
Experts					
*Intraclass κ*	0.74	0.78	0.77	0.68	0.62
*Specificity *	0.90	0.93	0.98	0.93	0.98
*Sensitivity *	0.88	0.88	0.79	0.89	0.61
Nonexperts					
*Intraclass κ*	0.54	0.59	0.62	0.52	0.36
*Specificity *	0.92	0.90	0.96	0.94	0.97
*Sensitivity *	0.73	0.72	0.67	0.54	0.33

## Abbreviations

BRS: Bolus residue scale
UES: Upper esophageal sphincter.

## References

[1] Dodds W. J., Logemann J. A., Stewart E. T. (1990). Radiologic assessment of abnormal oral and pharyngeal phases of swallowing. *American Journal of Roentgenology*.

[2] Eisenhuber E., Schima W., Schober E. (2002). Videofluoroscopic assessment of patients with dysphagia: pharyngeal retention is a predictive factor for aspiration. *American Journal of Roentgenology*.

[3] Omari T. I., Dejaeger E., Tack J., Vanbeckevoort D., Rommel N. (2012). An impedance-manometry based method for non-radiological detection of pharyngeal postswallow residue. *Neurogastroenterology & Motility*.

[4] Logemann J. A. (1998). *Evaluation and Treatment of Swallowing Disorders*.

[5] Dejaeger E., Pelemans W., Ponette E., Joosten E. (1997). Mechanisms involved in postdeglutition retention in the elderly. *Dysphagia*.

[6] Pearson W. G., Molfenter S. M., Smith Z. M., Steele C. M. (2013). Image-based measurement of post-swallow residue: the normalized residue ratio scale. *Dysphagia*.

[7] Hind J. A., Nicosia M. A., Roecker E. B., Carnes M. L., Robbins J. (2001). Comparison of effortful and noneffortful swallows in healthy middle-aged and older adults. *Archives of Physical Medicine and Rehabilitation*.

[8] Rosenbek J. C., Robbins J. A., Roecker E. B., Coyle J. L., Wood J. L. (1996). A penetration-aspiration scale. *Dysphagia*.

[9] Han T. R., Paik N.-J., Park J. W. (2001). Quantifying swallowing function after stroke: a functional dysphagia scale based on videofluoroscopic studies. *Archives of Physical Medicine and Rehabilitation*.

[10] Dyer J. C., Leslie P., Drinnan M. J. (2008). Objective computer-based assessment of valleculae residue: is it useful?. *Dysphagia*.

[11] Cohen J. (1960). A coefficient of agreement for nominal scales. *Educational and Psychological Measurement*.

[12] Landis J. R., Koch G. G. (1977). An application of hierarchical kappa-type statistics in the assessment of majority agreement among multiple observers. *Biometrics*.

[13] Farneti D. (2008). Pooling score: an endoscopic model for evaluating severity of dysphagia. *Acta Otorhinolaryngologica Italica*.

[14] Murray J., Langmore S. E., Ginsberg S., Dostie A. (1996). The significance of accumulated oropharyngeal secretions and swallowing frequency in predicting aspiration. *Dysphagia*.

[15] Stoeckli S. J., Huisman T. A. G. M., Seifert B., Martin-Harris B. J. W. (2003). Interrater reliability of videofluoroscopic swallow evaluation. *Dysphagia*.

[16] Omari T. I., Dejaeger E., Van Beckevoort D. (2011). A novel method for the nonradiological assessment of ineffective swallowing. *The American Journal of Gastroenterology*.

